# Loss of the Synuclein Family Members Differentially Affects Baseline- and Apomorphine-Associated EEG Determinants in Single-, Double- and Triple-Knockout Mice

**DOI:** 10.3390/biomedicines10123128

**Published:** 2022-12-04

**Authors:** Vasily Vorobyov, Alexander Deev, Iuliia Sukhanova, Olga Morozova, Zoya Oganesyan, Kirill Chaprov, Vladimir L. Buchman

**Affiliations:** 1 School of Biosciences, Sir Martin Evans Building, Cardiff University, Museum Avenue, Cardiff CF10 3AX, UK; 2 Institute of Cell Biophysics, Russian Academy of Sciences, 142290 Pushchino, Russia; 3 Institute of Theoretical and Experimental Biophysics, Russian Academy of Sciences, 142290 Pushchino, Russia; 4 Institute of Physiologically Active Compounds, Russian Academy of Sciences, 142432 Chernogolovka, Russia; 5 Center of Pre-Clinical and Clinical Studies, Belgorod State National Research University, 308015 Belgorod, Russia; 6 International School “Medicine of the Future”, I.M. Sechenov First Moscow State Medical University (Sechenov University), 119991 Moscow, Russia

**Keywords:** synucleins, dopamine, electroencephalogram, frequency spectrum

## Abstract

Synucleins comprise a family of small proteins highly expressed in the nervous system of vertebrates and involved in various intraneuronal processes. The malfunction of alpha-synuclein is one of the key events in pathogenesis of Parkinson disease and certain other neurodegenerative diseases, and there is a growing body of evidence that malfunction of other two synucleins might be involved in pathological processes in the nervous system. The modulation of various presynaptic mechanisms of neurotransmission is an important function of synucleins, and therefore, it is feasible that their deficiency might affect global electrical activity detected of the brain. However, the effects of the loss of synucleins on the frequency spectra of electroencephalograms (EEGs) have not been systematically studied so far. In the current study, we assessed changes in such spectra in single-, double- and triple-knockout mice lacking alpha-, beta- and gamma-synucleins in all possible combinations. EEGs were recorded from the motor cortex, the putamen, the ventral tegmental area and the substantia nigra of 78 3-month-old male mice from seven knockout groups maintained on the C57BL/6J genetic background, and 10 wild-type C57BL/6J mice for 30 min before and for 60 min after the systemic injection of a DA receptor agonist, apomorphine (APO). We found that almost any variant of synuclein deficiency causes multiple changes in both basal and APO-induced EEG oscillation profiles. Therefore, it is not the absence of any particular synuclein but rather a disbalance of synucleins that causes widespread changes in EEG spectral profiles.

## 1. Introduction

The synuclein family comprises three closely related proteins, specifically alpha-, beta- and gamma-synucleins (*alpha*-syn, *beta*-syn and *gamma*-syn), that are widely expressed in the nervous system of vertebrates, where they are involved in multiple intraneuronal processes, particularly those that are associated with synaptic neurotransmission [1,2]. *Alpha*-syn has been originally identified as a protein highly enriched in presynaptic terminals in various brain areas, where it is commonly colocalised with *beta*-syn [3]. The involvement of *alpha*-syn and *beta*-syn in chemical, particularly dopaminergic, neurotransmission has been linked with their role in the molecular pathogenesis of Parkinson disease (PD) [4,5], dementia with Lewy bodies (DLBs) and certain other neurodegenerative diseases (so-called synucleinopathies) [6,7,8]. *Gamma*-syn is predominantly expressed in sensory neurons; nevertheless, it has been shown to induce cortical astrocyte proliferation with subsequent BDNF expression and release [9]. Furthermore, *gamma*-syn is able to modify *beta*-syn-membrane interaction [10] and control midbrain dopamine (DA) function [11].

Various animal models based on the expression of mutated forms or simply overexpression of these proteins (*alpha*-syn, in particular) have been developed that displayed neurological dysfunction, which supported a notion about the gain of function, i.e., pathological aggregation, as the main cause of neurodegeneration in these diseases [12,13]. However, to better understand pathological mechanisms triggered by malfunction of synucleins, their roles in normal brain physiology need to be clarified. Interpretations of results of such studies are complicated by a potential functional redundancy within the family due to the high similarity of amino acid sequences and overlapping expression patterns [14,15]. In the triple-knockout (TKO) mice, some physiological mechanisms (age dependent, in particular) were found to be affected to a greater extent than in single- or double-synuclein knockouts (KOs) [16,17,18]. These studies also demonstrated that although synucleins are not essential for basic synaptic functions, they are required for the stabilisation and maintenance of DA level in the mediatory system [19,20]. It has also been revealed that learning abilities in tasks requiring intact spatial and working memory are compromised in *alpha*-syn KO mice, indicating an important role of *alpha*-syn in cognitive processes [21,22]. At the same time, *gamma*-syn KO mice are characterised by increased orientational and exploratory behaviour, reduced state anxiety and enhanced fear memory [23,24].

Synaptic dysfunctions and an imbalance between coordinated activities of different brain structures are hypothesised to be the main cause of abnormal functioning of the diseased brain [25]. It is feasible to suggest that by modifying synaptic transmission the deficiency of synuclein family member(s) would disrupt the neuronal network functioning, accompanied by modifications of electrical oscillations in the affected neuronal circuits [26], thus disrupting their interaction [27]. Superimposed extracellular fields arising from synaptic transmembrane currents of neurons involved in these circuits form the electroencephalogram (EEG) [28]. Changes in EEG patterns have been shown to be associated with PD pathology both in patients [29,30] and in animal models of PD [31,32]. In several recent EEG studies of PD, substantial attention has been paid to neuronal networks within/between the cortical and subcortical brain areas [33,34] and to the role of DA transmission in the functioning of these networks [20,35,36]. However, we are still lacking detailed information on how changes in the composition of synuclein family members affect the DA system. This needs to be clarified given data demonstrating a potential role of neuronal/synaptic plasticity in the DA system associated with synucleins [19]. In a few EEG studies, associations between modifications of *alpha*-syn and changes in the brain electrical activity have been shown [37,38]. Thus, a deeper insight into the activities of different neuronal networks and their changes in different types of synuclein KO mice would be beneficial for obtaining a better understanding of the role of synuclein family members in healthy and neurodegeneration-affected nervous systems.

In this study, we recorded EEGs from the motor cortex (MC), the putamen (Pt) and the DA-producing brain regions (ventral tegmental area (VTA) and substantia nigra (SN)) of adult mice with all possible combinations of *alpha*-, *beta*-, and *gamma*-syn KOs, before and after the systemic injection of a DA receptor agonist, apomorphine (APO). Genotype-specific and brain area–specific differences between various synuclein KO and control wild-type (WT) mice were revealed in the frequency spectra of both baseline and APO-evoked EEG from these brain areas.

## 2. Materials and Methods

### 2.1. Experimental Animals

In this study, 3-month-old male mice with different combinations of *alpha* B6(Cg)-Snca ^tm1.2Vlb^, *beta* B6(Cg)-Sncb ^tm1Sud^ and *gamma* B6(Cg)-Sncg ^tm1Vlb^ synuclein knockouts, all maintained on the C57BL/6J genetic background [39,40] and where WT mice originated from the same breeding programme, were used. Overall, eight cohorts of mice were compared: WT (A+B+G+, n = 10), ABG-KO (A-B-G-, n = 8), AG-KO (A-B+G-, n = 10), B-KO (A+B-G+, n = 11), A-KO (A-B+G+, n = 12), BG-KO (A+B-G-, n = 13), G-KO (A+B+G-, n = 11) and AB-KO (A-B-G+, n = 13).

Up to the age of 2 months, animals were housed in groups of five per cage, and thereafter, each of them was kept for 1 month in an individual cage. Mice were housed in a standard environment (12 h light/dark cycle, 22–25 °C RT, 50%–55% relative humidity) with food and water ad libitum. The procedures were carried out in accordance with the “Guidelines for accommodation and care of animals. Species-specific provisions for laboratory rodents and rabbits” (GOST 33216-2014), in compliance with the principles enunciated in the Directive 2010/63/EU on the protection of animals used for scientific purposes and approved by the local Institute Ethics Review Committee (protocol № 48, 15.01.2021). All efforts were made to minimise the number of the animals and their suffering. All mice were genotyped using a PCR analysis of DNA obtained from the ear biopsy, as described elsewhere [20].

### 2.2. Implantation of Electrodes and EEG Recording

After 1 month of adaptation to the individual cage, each mouse was anesthetised with the subcutaneous (s.c.) injection of a combination of dissolved tiletamine/zolazepam (Zoletil^®^, Virbac, Carros, France) and xylazine solution (Rometar^®^, Bioveta, Ivanovice na Hané, Czech Republic) at doses of 25 mg/kg and 2.5 mg/kg, respectively. Four recording electrodes were implanted into the left MC and Pt (MC and Pt; AP: +1.1 mm anterior to bregma; ML: ±1.5 mm lateral to midline; DV: −0.75 and −2.75 mm depths from skull surface, respectively), into the left VTA (AP: −3.1, ML: −0.4, DV: −4.5) and into the right SN (AP: −3.2, ML: +1.3, DV: −4.3) [41] (DV was measured from the skull surface). Within brain areas analysed in this study, the opposite hemisphere for SN was chosen, first because of its proximity to VTA, which meant we could not exclude possible mutual damage during electrode implantation in the same hemisphere. Second, it is well known that the contralateral SN is the dominant source of DA in the opposite hemisphere. Custom-made electrodes were constructed from two varnish-insulated nichrome wires (100 µm diameter) glued together (3M Vetbond^TM^ Tissue Adhesive, St. Paul, MN, USA) with 100 µm tips, free from insulation. Thus, the electrodes were sufficiently inflexible and had higher effective surface–volume ratio than a monowire electrode of a 200 µm diameter. The reference and ground electrodes (stainless steel wire, 0.4 mm in diameter) were placed symmetrically into the caudal cavities behind the cerebrum (AP: −5.3, ML: ±1.8, DV: −0.5). All electrodes were positioned using a computerised 3D stereotaxic StereoDrive (Neurostar, Tübingen, Germany), fixed to the skull with dental cement and soldered to a dual row socket connector (Sullins Connector Solutions, San Marcos, CA, USA). Each of nichrome wires was soldered to one of the connector’s pins. After electrode implantation, animals were housed individually for the recovery, followed by the experimental sessions. The postmortem verification of the electrode tip location included a preliminary anodal current (80–100 µA, 1 s) coagulation of the adjacent tissue and extirpation of the brain. The brains were fixed in Carnoy’s (60% ethanol, 30% chloroform, 10% glacial acetic acid) at 4 °C overnight following dehydration in alcohol series and embedding in paraffin blocks (see details in [14,42]). Furthermore, 8 μm thick coronal brain sections were cut using Leica Biosystems (Deer Park, IL, USA) microtome and mounted onto poly-L-lysine–coated slides as described previously [18]. One of five slides from each of eight series was stained with hematoxylin and eosin. Electrode positions in Pt and MC were visualised without additional immunostaining, whereas for those in SN and VTA, an adjacent slide was stained with antibodies against tyrosine hydroxylase (TH, mouse monoclonal antibody, clone TH-2, Sigma, diluted 1:1000) and secondary Goat anti-mouse IgG (H+L) highly cross-adsorbed second antibodies (Alexa Fluor 488, Thermo A11029 diluted 1:1000) as described previously [43]. The borders of SN and VTA on histological sections were outlined using the atlas of TH-positive cells distribution [44]. Representative images demonstrating coagulated tissues at the position of electrode tip either in the SN region or in the VTA region are shown in the Supplementary Figure S1.

Effective electrode targeting of the chosen brain areas was based on a precise measurement of the bregma and lambda coordinates and the providing of coordinates corrections for individual brain areas given that the value that was used for the preparation of the stereotaxic atlas [41] was equal to 4.2 ± 0.25 mm. In several cases, when the electrolytic marker was relatively enlarged, the electrode tip position was assigned to the point where the effect of electrolysis within the coagulated area was maximal.

Three days after electrode implantation, each mouse was adapted for four days (1 h/day) to both an experimental cage (Perspex, 15 cm × 17 cm × 20 cm) in an electrically shielded chamber and to a cable (five 36-gauge wires, Plexon Inc, Dallas, TX, USA) plugged into a digital Neuro-MEP amplifier (Neurosoft Ltd., Ivanovo, Russian Federation). On day 8, a baseline EEG was recorded for 30 min, starting 20 min after placing the animal into the box. EEG recordings were continued for 60 min after the s.c. injection of either saline (control) or, on the next day, apomorphine (APO, Sigma, Milan, Italy), at a dose of 1.0 mg/kg. To minimise the effect of oxidation, only freshly dissolved APO was used. All experiments were performed from 9:00 a.m. to 6:00 p.m. in daylight combined with an artificial light source, keeping illumination at a relatively stable level.

### 2.3. Computation of EEG Frequency Spectra

Monopolar EEG signals measured between the active and reference electrodes were amplified, filtered (0.1–35 Hz) and sampled (1 kHz) online by using the amplifier and kept in an operational computer for further analysis. The frequency spectra of successive 12 s EEG epochs were studied using a modified version of period-amplitude analysis [45], which, in contrary to the Fourier transform, was not affected by the well-known nonstationary nature of the EEG signals. The absolute values of the half-wave amplitudes with periods/frequencies in each of selected narrow EEG frequency sub-bands were summed, followed by their normalisation to the summarised values. The programme allowed both the automatic and the manual rejection of EEG fragments containing artefacts and electrographic seizures. However, artefacts appeared seldom, because of tight connections in the recording cable sockets and insertion of the cable into a thin, flexible, grounded silvered shield, to protect EEGs against so-called capacity artefacts. In this study, 25 sub-bands in the 0.48–31.5 Hz range were analysed: 0.48–0.53 (0.5), 0.83–0.92 (0.9), 1.20–1.33 (1.3), 1.59–1.76 (1.7), 1.99–2.20 (2.1), 2.42–2.67 (2.5), 2.86–3.17 (3.0), 3.34–3.69 (3.5), 3.83–4.24 (4.0), 4.36–4.82 (4.6), 4.92–5.44 (5.2), 5.52–6.10 (5.8), 6.17–6.82 (6.5), 6.87–7.59 (7.2), 7.62–8.43 (8.0), 8.45–9.34 (8.9), 9.37–10.36 (9.9), 10.40–11.49 (10.9), 11.56–12.77 (12.2), 12.90–14.26 (13.6), 14.49–16.01 (15.3), 16.43–18.16 (17.3), 18.93–20.93 (19.9), 22.47–24.83 (23.6) and 28.50–31.50 (30.0). The sub-bands are marked in figures by their centre (mean) frequency values (see in brackets above). The final analyse was performed for “classical” EEG bands: *delta 1* (0.5–1.7 Hz), *delta 2* (2.1–3.5 Hz), *theta* (4.0–8.0 Hz), *alpha* (8.9–12.2 Hz), *beta 1* (13.6–17.3) and *beta 2* (19.9–30.0).

The frequency spectra of 12 s EEG epochs were individually averaged for every successive 10 min interval for each mouse, followed by the separate averaging of the individual values in WT and in each of the KO groups. The differences in the averaged spectra of baseline EEG in different groups allow the evaluation of a role of the synucleins (and/or their lack) in the modification of the EEG frequency spectra, whereas the differences in EEG effects of APO are expected to be associated with specific modifications of the DA system by synucleins.

### 2.4. Statistics

Differences in the “classical” frequency ranges of the averaged EEG spectra from each brain area were evaluated by two-way ANOVA for repeated measures between different cohorts of animals in the baseline 30 min interval and for 60 min after APO (vs. saline) injection. For multiple comparisons, a Bonferroni post hoc test was employed. The group data were expressed as the means ± SEM; differences were considered significant at *p* < 0.05. For two-way ANOVA, STATISTICA 10 (StatSoft, Inc., Tulsa, OK, USA) was used.

## 3. Results

### 3.1. Baseline EEG

During baseline EEG recordings, mice with knockouts of synuclein genes behaved similarly to WT control mice: displayed intensive exploration of the experimental box with stochastically scattered sleep-like bouts.

**Figure 1:**
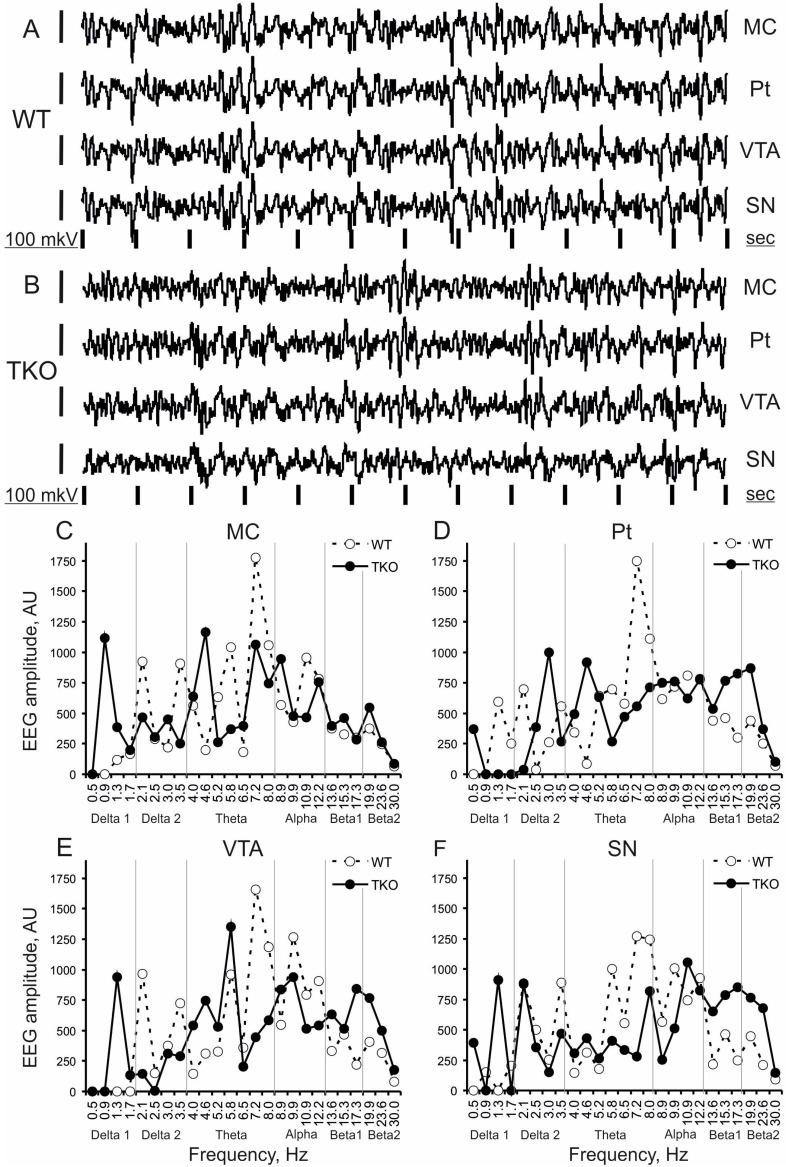
Baseline EEG fragments and their frequency spectra in a 3-month-old triple-knockout mouse vs. a wild-type littermate. Typical patterns in 12 s fragments of baseline EEG in wakeful and behaviourally active wild-type (WT) and triple (*alpha-, beta-, and gamma-*synucleins) knockout (TKO) mice ((**A**) and (**B**), respectively) and their frequency spectra (**C**–**F**) in the motor cortex (MC), putamen (Pt), ventral tegmental area (VTA) and substantia nigra (SN). On A and B, time calibration is 1 s and amplitude calibration is 100 µV. On (**C**–**F**), abscissa is a frequency sub-band marked with its mean value, in hertz ordinate is the summed amplitudes of EEG in each of the 25 sub-bands, normalised to a sum of all amplitude values, in arbitrary units. Vertical grey lines separate “classical” EEG frequency bands: *delta 1*, *delta 2, theta*, *alpha*, *beta 1*, *and beta 2*.

**Figure 2:**
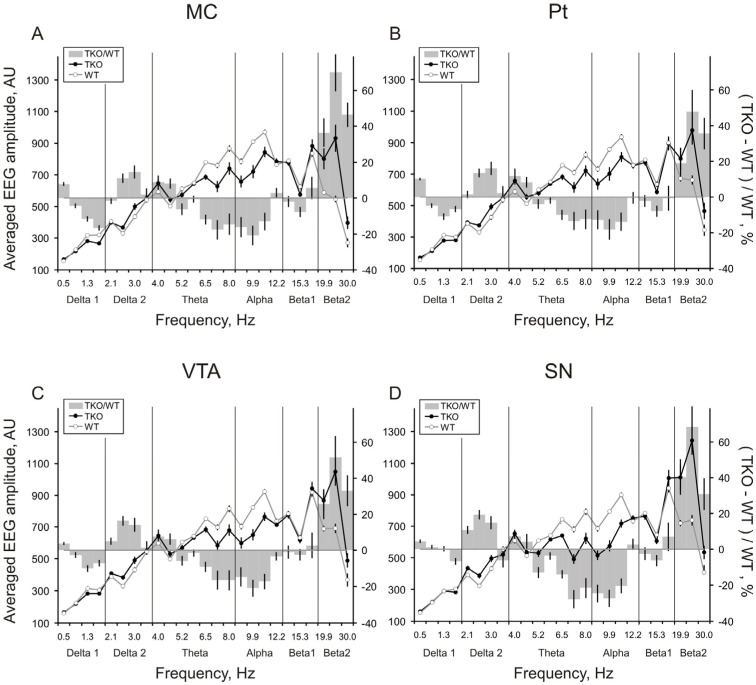
Averaged baseline EEG spectra in triple-knockout mice vs. wild-type littermates. Averaged amplitude-frequency spectra of 12 s baseline EEG fragments recorded from the motor cortex (**A**), putamen (**B**), VTA (**C**) and SN (**D**) for 30 min in wild-type (WT, n = 10) and triple (*alpha-, beta-, and gamma-*synucleins) knockout (TKO, n = 8) mice (dashed and solid lines, respectively) and spectral ratios (grey bars) between the groups (TKO/WT) in %. Abscissa is a frequency sub-band marked with its mean value, in hertz; the left ordinate is summed absolute values of EEG amplitudes in each of 20 sub-bands, normalised to sum of all amplitude values, in arbitrary units; and the right ordinate is a ratio of the EEG amplitudes, calculated as (TKO-WT) / WT, in %. Vertical lines are ± 1 SEM. Vertical grey lines separate “classical” EEG frequency bands: *delta 1*, *delta 2, theta*, *alpha*, *beta 1*, *and beta 2*.

Baseline EEGs in WT (A+B+G+) and TKO (A-B-G-) mice (Figure 1A and B, respectively) were characterised by patterns of relatively slow oscillations of 6–12 Hz, more powerfully expressed in WT mouse. In contrast, the fastest EEG activity of 19.9–30 Hz predominated in Pt, VTA, and SN in a TKO mouse. These EEG patterns were represented in their frequency spectra by higher peaks in the *upper theta*-*alpha* range and *beta 2* band in WT and TKO mice, respectively (Figure 1C–F). These differences between the groups were stable in EEG spectra averaged over consecutive 10 min intervals and, thus, were evidently observed in the spectral profiles that characterised the whole (30-min) baseline period (Figure 2).

**Figure 3:**
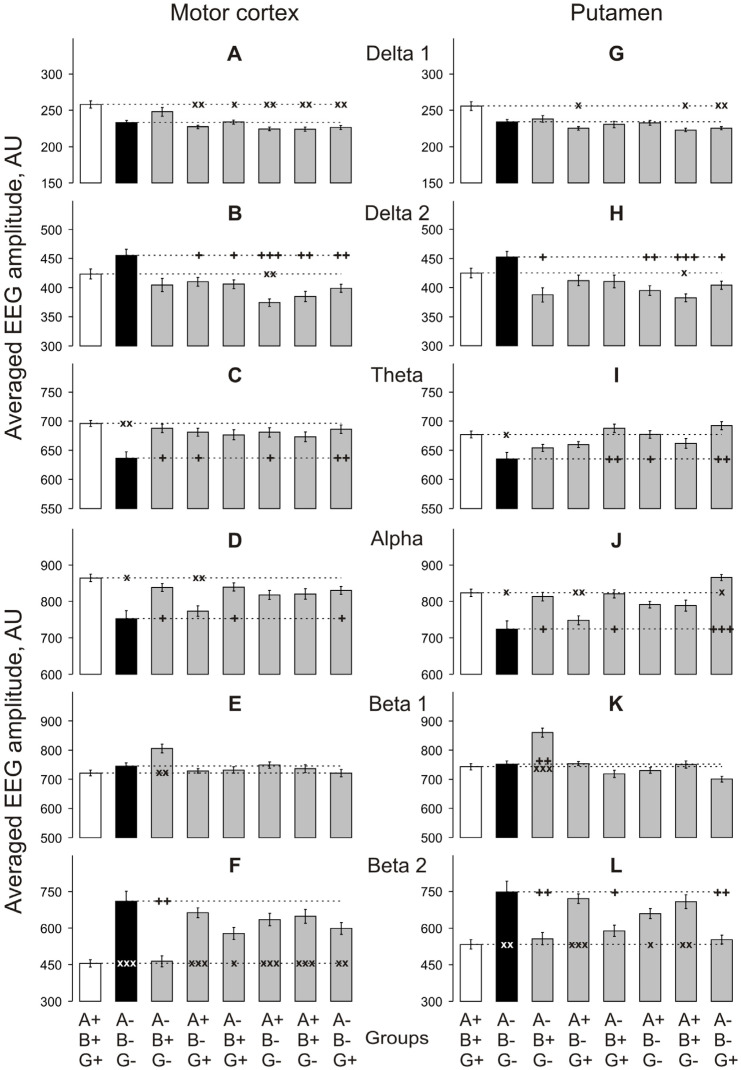
Relations between averaged amplitudes in “classical” frequency bands of 12 s baseline EEG fragments recorded from the motor cortex (**A**–**F**) and putamen (**G**–**L**) for 30 min in knockout mice of different types denoted on the horizontal axes. Ordinate is the averaged absolute values of EEG amplitudes in each of the “classical” bands, in arbitrary units (vertical lines are ±1 SEM). Horizontal dashed lines denote the values in wild-type and triple-knockout groups: x and + symbols denote significant two-way ANOVA differences from the wild-type and triple-knockout mice, respectively (one, two and three symbols denote *p* < 0.05, <0.01 and 0.001, respectively).

**Figure 4:**
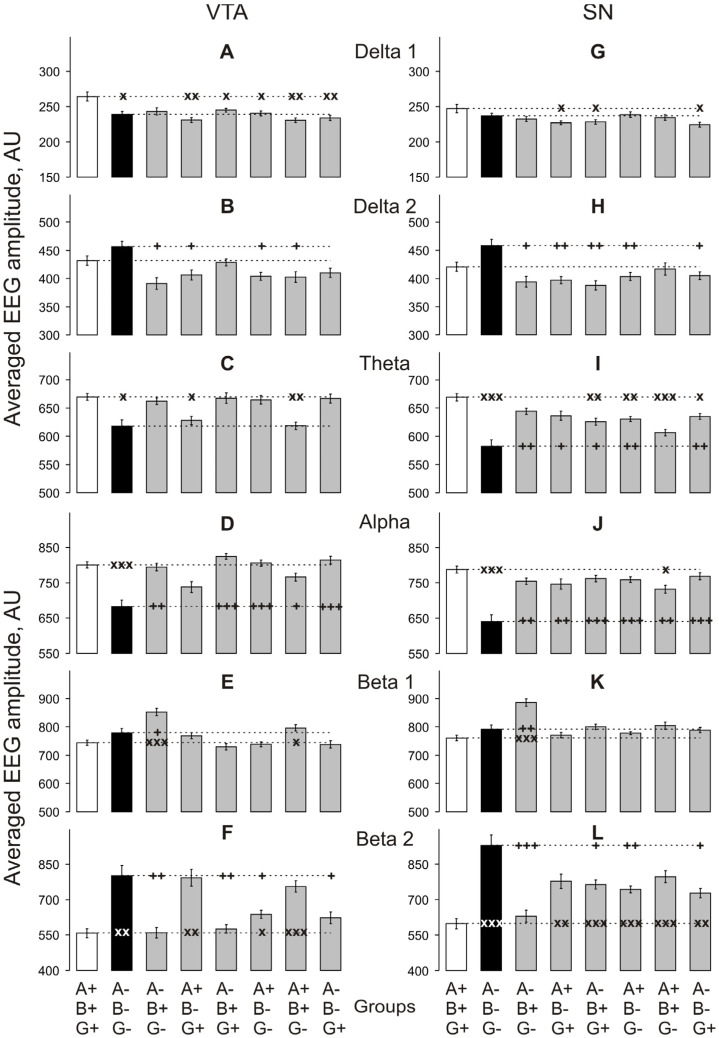
Relations between averaged amplitudes in “classical” frequency bands of 12 s baseline EEG fragments recorded from VTA (**A**–**F**) and SN (**G**–**L**) for 30 min in knockout mice of different types denoted on the horizontal axes. Ordinate is the averaged absolute values of EEG amplitudes in each of the “classical” bands, in arbitrary units (vertical lines are ±1 SEM). Horizontal dashed lines denote the values in wild-type and triple-knockout groups: x and + symbols denote significant two-way ANOVA differences from the wild-type and triple-knockout mice, respectively (one, two and three symbols denote *p* < 0.05, < 0.01 and 0.001, respectively).

**Figure A1:**
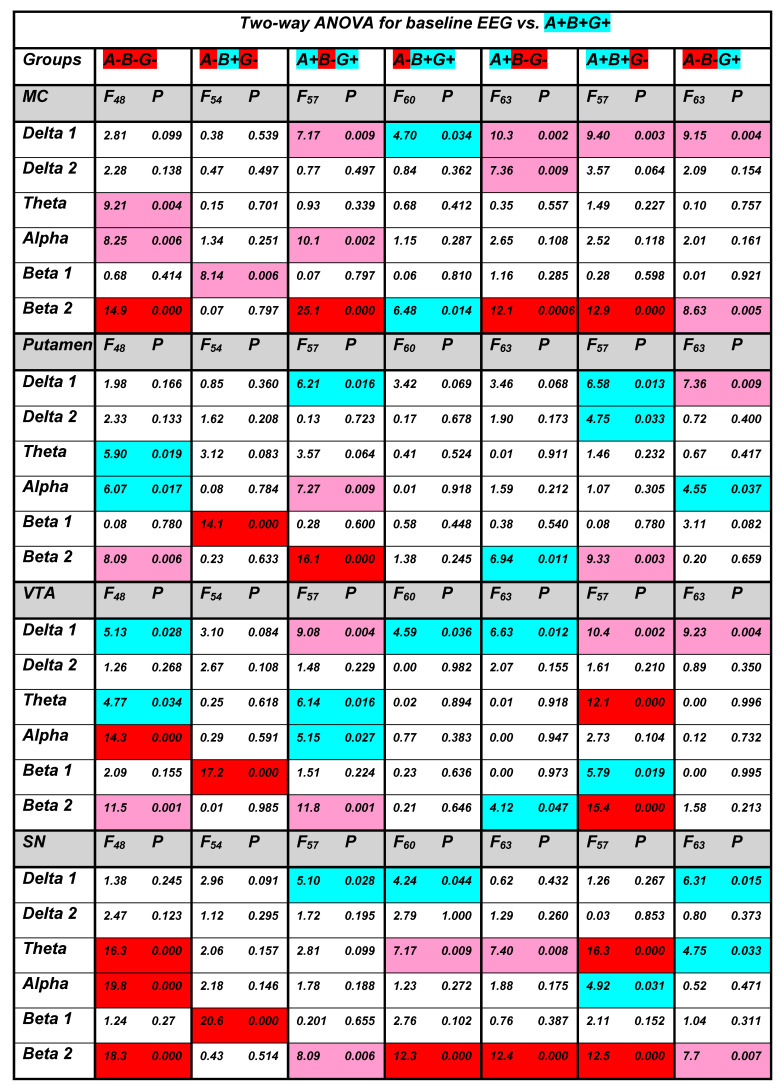
Results of a two-way ANOVA analysis of baseline changes in EEG recordings from four brain areas of synuclein KO mice vs. WT (A+B+G+) mice. Data with *p* < 0.05 are highlighted blue, *p* < 0.01 highlighted pink and *p* < 0.001 highlighted red.

During this period, baseline EEG activity in MC in TKO vs. WT mice (Figure 3A–F) was significantly suppressed in both *theta* and *alpha* bands (Figure 3C,I; two-way ANOVA: F_1,48_ = 9.2 and 8.2, respectively, *p* < 0.01 for both) and enhanced in *beta 2* band (Figure 3F; two-way ANOVA: F_1,48_ = 14.9, *p* < 0.001). In Pt (Figure 3G–L), the EEG differences in the *theta, alpha* and *beta 2* bands were similar to those in MC (two-way ANOVA: F_1,48_ = 5.9, 6.1 and 8.1, *p* < 0.05 for both, and < 0.01, respectively). In VTA (Figure 4A–F) and SN (Figure 4G–F), significant differences between TKO and WT mice were observed in the same frequency ranges (F_1,48_ = 4.8, 14.3 and 11.5, *p* < 0.05, 0.001 and 0.01, respectively, for VTA, and F_1,48_ = 16.3, 19.8 and 18.3, respectively, *p* < 0.001 for SN). All two-way ANOVA evaluations vs. WT group are presented in Appendix A Figure A1.

To assess whether the changes in the EEG spectra observed in the brain areas of TKO mice developed only as the result of the absence of all three synucleins or whether the depletion of certain family member(s) could be sufficient for the development of the same changes, we measured these spectra in the brain areas of mice lacking one or two synucleins in all six possible combinations.

Changes in *delta 1* activity were found to be quite diverse between genotypes and brain regions when compared with WT mice, although this activity was lower in all cases when the difference was significant (Figure 3A,G and Figure 4A,G). Conversely, *delta 2* activity that was not affected in any of studied brain areas of TKO mice showed no significant changes in the SN and VTA of mice of all single- and double-KO genotypes, whereas a decrease was observed in the Pt of A + B+G- and MC of A + B-G- mice when compared with WT mice. The *theta* activity that was depressed in the TKO vs. WT group in all brain areas (Figure 3C,I and Figure 4C,I) appeared to be unaffected in the MC and Pt of all other KO-genotype mouse groups (Figure 3C,I) but was depressed in the VTA of mice lacking either only *beta*-syn (A+B-G+) or only *gamma*-syn (A+B+G-) (Figure 4C,I). In the SN, an even-more-diverse pattern of the *theta* activity was observed with its depression in mice either lacking *alpha*-syn (A-B+G+) or expressing this protein in the absence of the other two synucleins (A+B-G-)—and either lacking *gamma*-syn (A+B+G-) or expressing this protein in the absence of the other two synucleins (A-B-G+) (Figure 4C,I). The *alpha* activity that was also depressed in the TKO vs. WT group in all brain areas (Figure 3D,J and Figure 4D,J) showed no such changes in most of the KO genotypes and brain areas, except for the MC and Pt of mice lacking *beta*-syn (A+B-G+) (Figure 3D,J) and the SN of mice lacking *gamma*-syn (A+B+G-) (Figure 4J). The *beta 1* activity was characterised by the most synuclein-independent pattern of activity throughout brain areas and genotypes, but it was consistently enhanced in all brain areas of mice expressing only *beta*-syn (Figure 3E,K and Figure 4E,K). In contrast, these mice showed no changes in *beta 2* activity in all four studied brain areas when compared with WT mice (Figure 3F,L and Figure 4F,L). Similarly, no changes in *beta 2* activity were found in the MC and VTA of mice either lacking *alpha*-syn (A-B+G+) or expressing this protein in the absence of the other two synucleins (A+B-G-), whereas all other combinations of KO genotypes, including TKO, and brain area enhanced *beta 2* activity (Figure 3F,L and Figure 4F,L).

### 3.2. Apomorphine Effects

In both WT and synuclein KO mice, APO initiated stereotyped behaviour, i.e., short-lasting freezing followed by uninterrupted licking of the floor and raising of the erected tail for about 30 min after injection. Also, occasional short sleep-like bouts were observed during this period in all APO-treated groups of mice.

#### 3.2.1. Apomorphine vs. Saline

**Figure 5:**
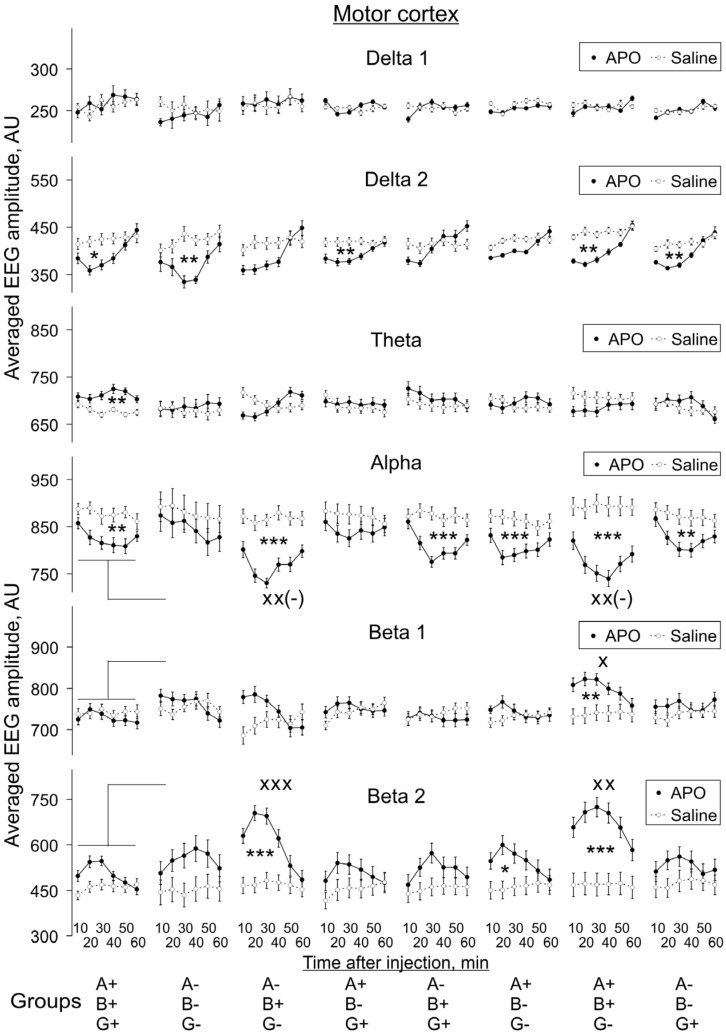
The evolution of apomorphine (APO, 1.0 mg/kg, s.c.) vs. saline effects in “classical” frequency bands of 12 s fragments of EEG from the motor cortex averaged for consecutive 10 min intervals (abscissa) in knockout mice of different types denoted on the horizontal axes. Ordinate is the averaged absolute values of EEG amplitudes in each of the “classical” bands, in arbitrary units (vertical lines are ±1 SEM), obtained in experiments with saline and APO (grey and black lines, respectively) in each group. Differences between baseline EEGs in knockout and wild-type mice were used to normalise APO effects in knockout groups to those in control. Symbols * and x denote significant differences in APO vs. saline and knockout mice vs. wild-type control, respectively (one, two and three symbols denote *p* < 0.05, <0.01 and 0.001, respectively). Symbol x(-) denotes the significant enhancement of the APO suppressive effect, for clarity.

In MC (Figure 5), APO significantly suppressed *delta 2* and *alpha* activities and enhanced the *theta* in WT (A+B+C+) mice (two-way ANOVA: F_1,108_ = 4.3, 9.8 and 6.7, *p* = 0.04, 0.002 and 0.009, respectively). In TKO (A-B-G-) mice, APO produced *delta 2* suppression in MC (two-way ANOVA: F_1,84_ = 9.9, *p* = 0.002), whereas the A-B+G- group was characterised by *alpha* suppression and *beta 2* enhancement (two-way ANOVA: F_1,108_ = 27.5 and 14.7, respectively, *p* < 0.001 for both). In A+B-G+ mice, APO suppressed *delta 2* activity (two-way ANOVA: F_1,120_ = 9.8, *p* = 0.002), whereas in A-B+G+ mice, it produced significant attenuation of *alpha* activity (two-way ANOVA: F_1,132_ = 14.9, *p* < 0.001). In the A+B-G- group, *alpha* suppression and *beta 2* enhancement were observed (two-way ANOVA: F_1,144_ = 11.4 and 5.8, *p* < 0.001 and = 0.02, respectively). The mice most sensitive to APO were in the A+B+G- group, for which significant suppression of *delta 2* and *alpha* activities (two-way ANOVA: F_1,120_ = 9.2 and 31.2, *p* = 0.003 and < 0.001, respectively) and enhancement of *beta 1* and *beta 2* ones (two-way ANOVA: F_1,120_ = 9.2 and 25.0 *p* = 0.003 and < 0.001, respectively) were revealed.

**Figure 6:**
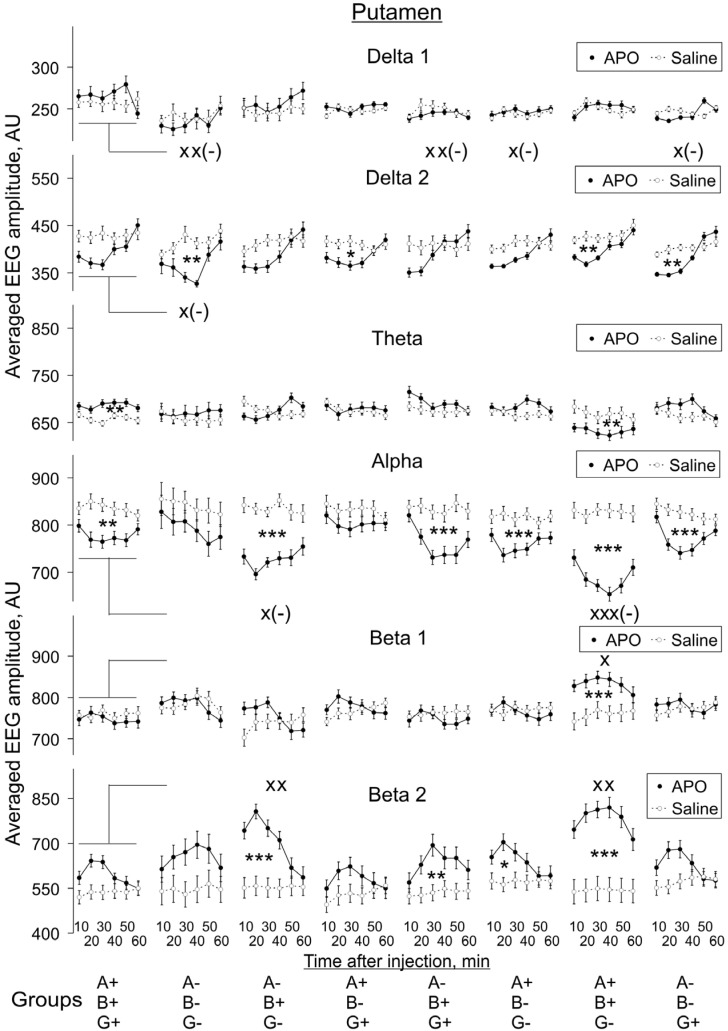
Evolution of apomorphine (APO, 1.0 mg/kg, s.c.) vs. saline effects in “classical” frequency bands of 12 s fragments of EEG from the putamen averaged for consecutive 10 min intervals (abscissa) in knockout mice of different types denoted on the horizontal axes. Ordinate is the averaged absolute values of EEG amplitudes in each of the “classical” bands, in arbitrary units (vertical lines are ±1 SEM), obtained in experiments with saline and APO (grey and black lines, respectively) in each group. Differences between baseline EEGs in knockout and wild-type mice were used to normalise the APO effects in knockout groups to those in control. Symbols * and x denote the significant differences in APO vs. saline and knockout mice vs. wild-type control, respectively (one, two and three symbols denote *p* < 0.05, <0.01 and 0.001, respectively). Symbol x(-) denotes the significant enhancement of the APO suppressive effect, for clarity.

In Pt (Figure 6), the distribution of significant differences after APO vs. saline injections was similar to that observed in MC, but in WT (A+B+G+) mice, *delta 2* suppression did not reach significant values (two-way ANOVA: F_1,108_ = 3.7, *p* = 0.056), whereas *beta 2* activity was significantly enhanced in the A-B+G+ group (two-way ANOVA: F_1,132_ = 7.5, *p* = 0.007), and *theta* activity was significantly supressed in the A+B+G- group (two-way ANOVA: F_1,120_ = 8.3, *p* = 0.005).

**Figure 7:**
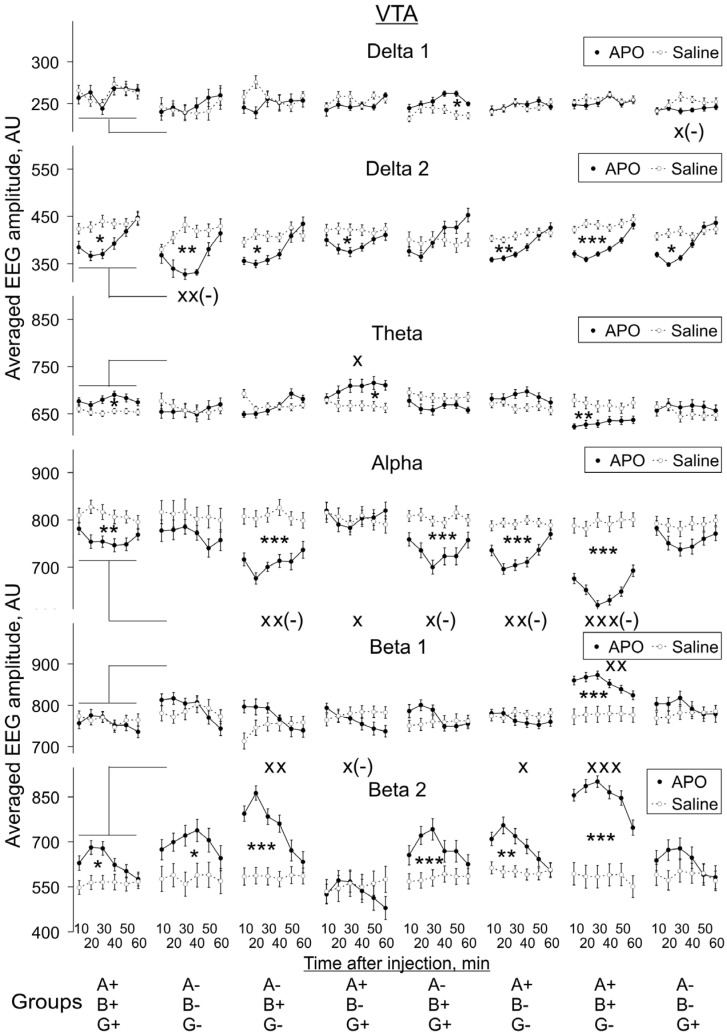
Evolution of apomorphine (APO, 1.0 mg/kg, s.c.) vs. saline effects in “classical” frequency bands of 12 s fragments of EEG from VTA averaged for consecutive 10 min intervals (abscissa) in knockout mice of different types. Ordinate is the averaged absolute values of EEG amplitudes in each of the “classical” bands, in arbitrary units (vertical lines are ±1 SEM), obtained in experiments with saline and APO (grey and black lines, respectively) in each group. Differences between baseline EEGs in knockout and wild-type mice were used to normalise the APO effects in knockout groups to those in control. Symbols * and x denote the significant differences of APO vs. saline and knockout mice vs. wild-type control, respectively (one, two and three symbols denote *p* < 0.05, <0.01 and 0.001, respectively). Symbol x(-) denotes the significant enhancement of the APO suppressive effect, for clarity.

All changes in EEG following APO administration observed in Pt and MC were also observed in the VTA of mice of corresponding genotypes (Figure 7), except for the lack of suppression of *alpha* activity in the A-B-G+ group (two-way ANOVA: F_1,144_ = 3.6, *p* = 0.059). In addition, in the A-B+G- and A+B-G- groups, *delta 2* activity was suppressed (two-way ANOVA: F_1,108_ = 4.1, *p* = 0.045, and F_1,144_ = 11.1, *p* = 0.001, respectively): in the A+B-G+ group, *theta* activity was increased (two-way ANOVA: F_1,120_ = 6.3, *p* = 0.014), as was *beta 2* activity in the WT and A-B-G- groups (two-way ANOVA: F_1,108_ = 5.1, *p* = 0.026, and F_1,84_ = 4.5, *p* = 0.038, respectively).

**Figure 8:**
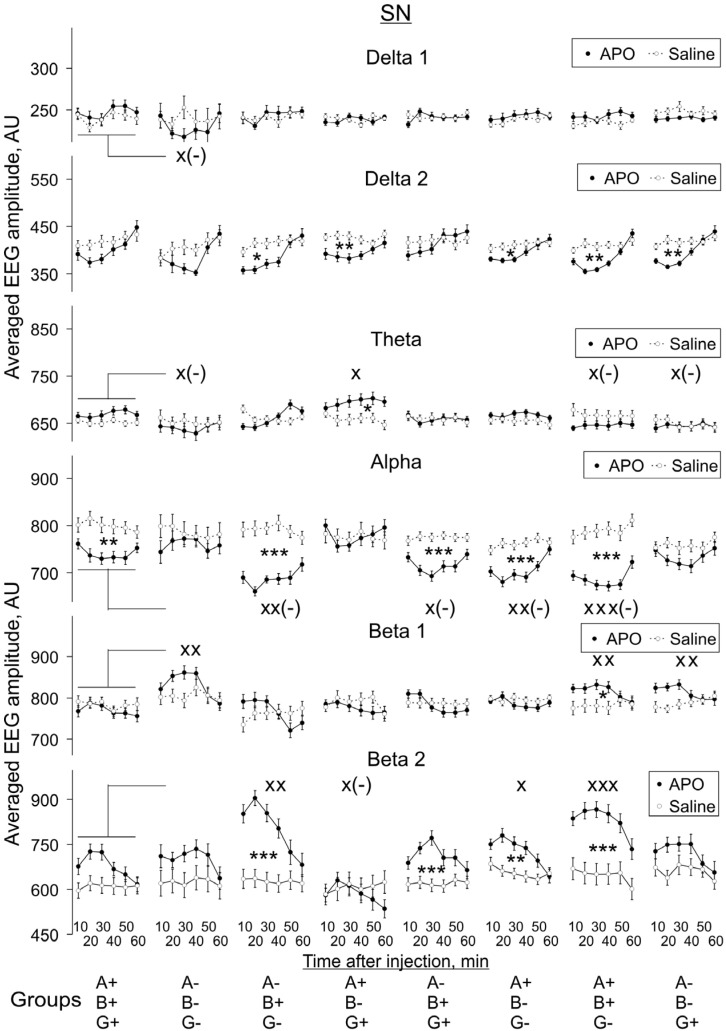
Evolution of apomorphine (APO, 1.0 mg/kg, s.c.) vs. saline effects in “classical” frequency bands of 12 s fragments of EEG from SN averaged for consecutive 10 min intervals (abscissa) in knockout mice of different types. Ordinate is the averaged absolute values of EEG amplitudes in each of the “classical” bands, in arbitrary units (vertical lines are ±1 SEM), obtained in experiments with saline and APO (grey and black lines, respectively) in each group. Differences between baseline EEGs in knockout and wild-type mice were used to normalise the APO effects in knockout groups to those in control. Symbols * and x denote the significant differences of APO vs. saline and knockout mice vs. wild-type control, respectively (one, two and three symbols denote *p* < 0.05, <0.01 and 0.001, respectively). Symbol x(-) denotes the significant enhancement of the APO suppressive effect, for clarity.

**Figure A2:**
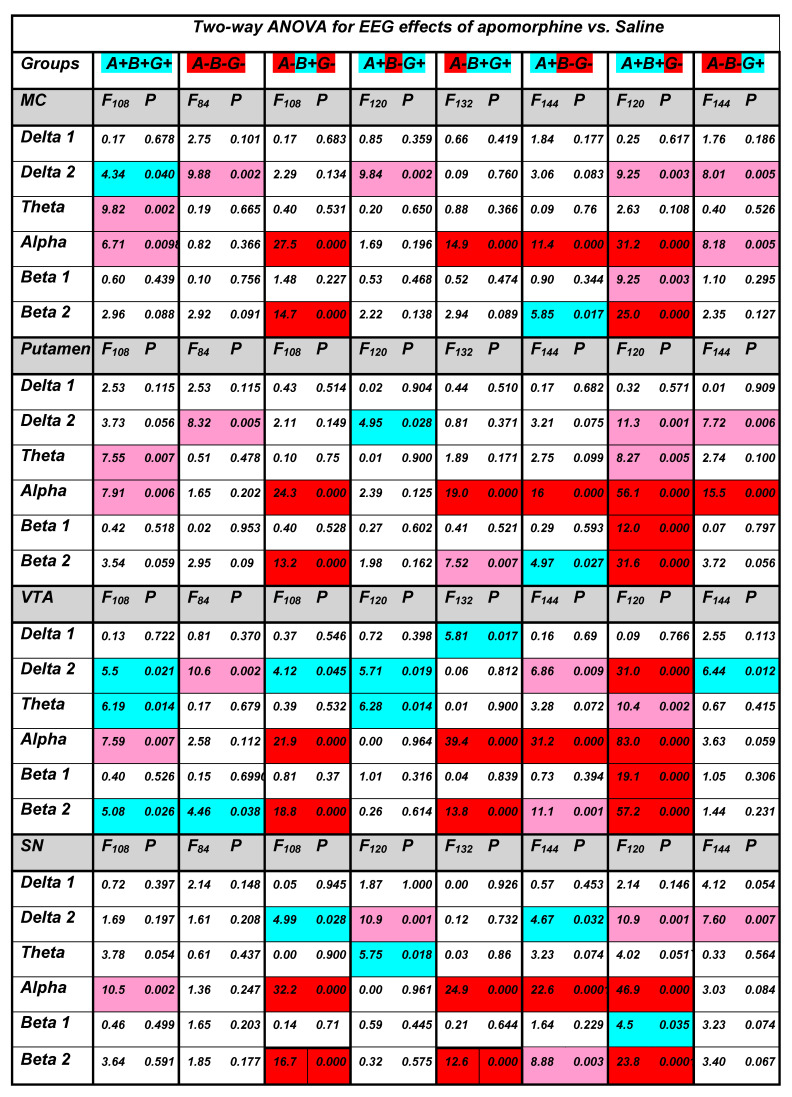
Results of a two-way ANOVA analysis of APO-induced changes in EEG recordings from four brain areas of synuclein KO mice and WT (A+B+G+) mice. Recoding from the same brain areas of mice injected with saline were used as controls. Data with *p* < 0.05 are highlighted blue, *p* < 0.01 highlighted pink and *p* < 0.001 highlighted red.

In SN the patterns of *alpha*, *beta 1* and *beta 2* activities following APO treatment were the same as those in Pt (Figure 7 and Figure 8), except for *alpha* activity in the A-B-G+ group, in which no significant difference was detected (two-way ANOVA: F_1,144_ = 3.0, *p* = 0.084). An increase of *theta* activity in SN was observed only in the A+B-G+ group (two-way ANOVA: F_1,120_ = 5.8, *p* = 0.018) and decreased *delta 2* activity in the same five groups as in VTA but not in the WT and A-B-G- groups (two-way ANOVA: F_1,108_ = 1.7, *p* = 0.197, and F_1,84_ = 1.6, *p* = 0.802, respectively). All two-way ANOVA evaluations vs. saline are presented in Appendix A Figure A2.

#### 3.2.2. APO Effects in Different Groups vs. Those in WT Mice

**Figure A3:**
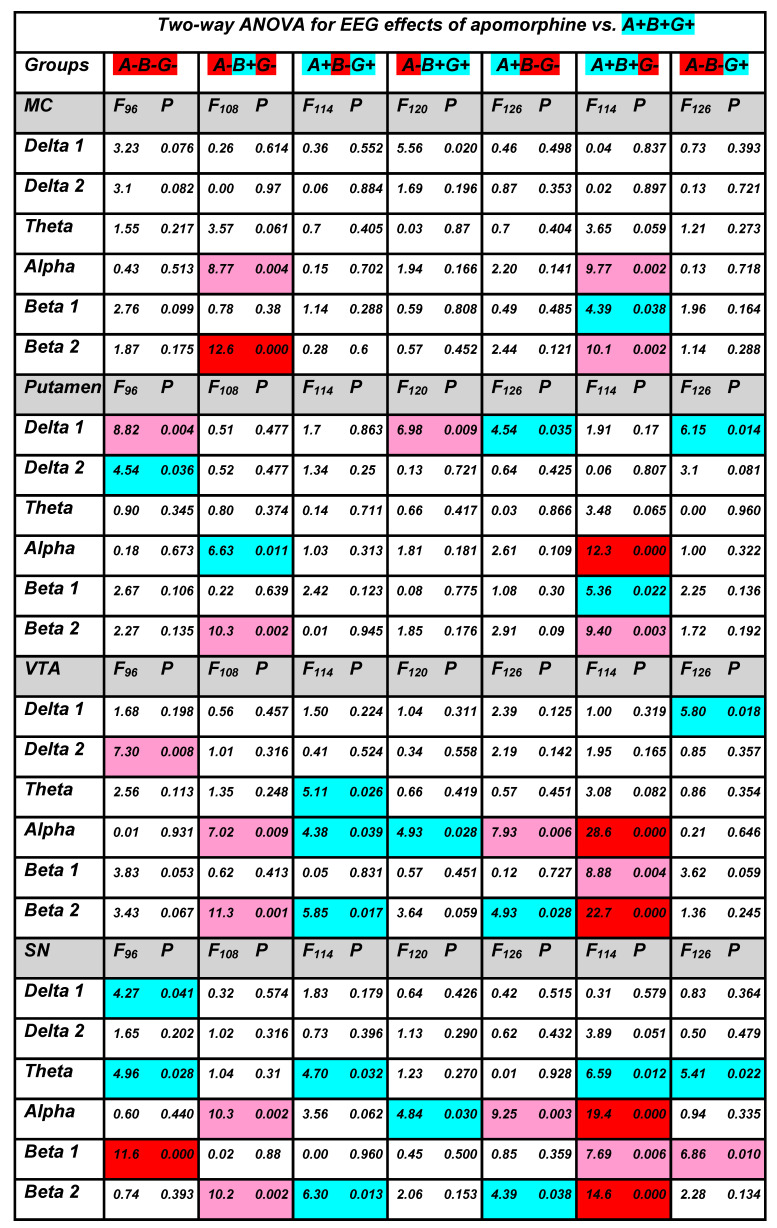
Results of a two-way ANOVA analysis of APO-induced changes in EEG recordings from four brain areas of synuclein KO mice vs. WT (A+B+G+) mice. Data with *p* < 0.05 are highlighted blue, *p* < 0.01 highlighted pink and *p* < 0.001 highlighted red.

We also compared APO-induced changes in EEG spectra in KO groups with those in the WT group, and these changes are also indicated in Figure 5, Figure 6, Figure 7 and Figure 8. The two-way ANOVA evaluations shown in Appendix A Figure A3 demonstrate the statistical significance of the differences in the degree of these changes, independently of whether APO injection induced a decrease or whether it induced an increase of a particular EEG band activity compared with saline injection.

The effect of animal genotype on APO-induced changes in EEG spectra was most profound in *gamma*-syn KO (A+B+G-) mice: a statistically significant decrease in the degree of changes was revealed for *alpha* activity and a statistically significant increase for *beta 1* and *beta 2* activities in all four studied brain areas. The effect on *alpha* and *beta 2* activity remained when both *gamma*-syn and *alpha*-syn synuclein were absent in double-KO animals (A-B+G-), but the effect on *beta 1* could not be seen anymore. The further removal of *beta*-syn abolished the effects of APO treatment on *alpha, beta 1* and *beta 2* activities in all four brain areas of TKO (A-B-G-) mice, with the exception of *beta 1* in SN (Figure 5, Figure 6, Figure 7 and Figure 8, Appendix A Figure A3).

In MC, the only detected APO-induced changes across synuclein KO genotypes were described above changes in *alpha* and *beta* activities, but all the other three studied brain areas were characterised by a more profound role of dopamine neurotransmission. Such changes were also observed for *delta 1* activity, particularly its decrease in Pt, and for *theta* activity, particularly in SN. A statistically significant decrease in *delta 2* activity was noticed only in the Pt and VTA of TKO (A-B-G-) mice (Figure 5, Figure 6, Figure 7 and Figure 8, Appendix A Figure A3).

## 4. Discussion

In this study, we have revealed the effects of all possible combinations of synuclein family members’ depletion (three single-, three double- and the triple-synuclein gene KOs) on *baseline* and *apomorphine-modified EEGs* recorded from the different brain areas of mice: motor cortex (MC), putamen (Pt), ventral tegmental area (VTA) and substantia nigra (SN).

Not surprisingly, across the studied brain area, the most frequent changes in baseline EEG spectral profiles, when compared with those in WT mice, were observed in the absence of all three synucleins, i.e., in TKO mice (schematically illustrated in Supplementary Figure S2), although no changes in *delta 2* or *beta 1* spectra were detected in these animals. This is consistent with substantial changes in synaptic morphology (e.g., decreased presynaptic terminal area of CA3 excitatory synapses) and activity (e.g., changes in the amplitude of the field excitatory postsynaptic potentials in the hippocampus) previously observed in TKO mice [16]. Interestingly, among single- and double-synuclein KO genotypes, the pattern of changes seen in mice lacking only *gamma*-syn was the most similar to the pattern seen in TKO mice, particularly in basal ganglia, SN and VTA (Supplementary Figure S2). This is in line with the recently obtained evidence that *gamma*-syn transcription in DA neurons modifies DA mediation in the brain [11]. However, this similarity of patterns gets lost in the absent of an additional member of the synuclein family, i.e., in *beta*-syn/*gamma*-syn and particularly in *alpha*-syn/*gamma*-syn double-KO mice (Supplementary Figure S2). Moreover, *gamma*-syn on its own, i.e., in *alpha*-syn/*beta*-syn double-KO mice, is not able to restore the WT pattern. It is *beta*-syn that singularly can normalise average EEG amplitudes at all but *beta 1* frequencies in *alpha*-syn/*gamma*-syn double-KO mice (Supplementary Figure S2), which is consistent the ability of *beta*-syn to potentiate neurotransmitter uptake by synaptic vesicles in the absence of other synucleins [6] and would suggest a key role of this protein in the regulation of EEG oscillations. Yet the absence of *beta*-syn either singularly or in combination with another synuclein (i.e., in *beta*-syn/*alpha*-syn or *beta*-syn/*gamma*-syn double-KO mice) causes changes in only some oscillation frequencies in certain brain areas compared with corresponding areas in the brain of WT mice. Thus, it is not the absence of any particular synuclein but rather a disbalance of synucleins that causes widespread changes in EEG spectral profiles.

Another observation that would need further investigation is that independently of the genotype and the brain area, the disbalance of synucleins always alters the vector of *beta 1* and *beta 2* level changes towards their enhancement but for other, higher frequencies, towards their suppression. A link between alterations in EEG oscillations, particularly the elevation of low-frequency *beta* oscillations, with motor impairment and neurodegeneration in PD patients and animal models of the disease has been reported in multiple studies [46,47,48]. Moreover, the suppression of *beta* oscillations correlates with the positive effects of symptomatic treatments of PD by levodopa or deep brain stimulation (DBS) [49,50,51]. We found that oscillations recorded from SN and VTA areas appeared to be substantially affected in all synuclein KO mice. Together with the aforementioned enhancement of *beta* oscillations in these mice, which resembles changes in *beta* frequencies EEG recordings in PD patients, these observations suggested that the modulation of dopaminergic neurotransmission in mice lacking certain synucleins might have specific effects on EEG recordings, particularly from these two brain areas. To test this, we treated WT and synuclein KO mice with APO, a DA agonist of several DA receptors and thus an activator of DA signalling.

APO treatment causes fewer changes in EEG oscillations in TKO mice than in all other studied genotypes, whereas the most changes were observed in mice lacking *gamma*-syn in the presence of one or two other members of the family (Supplementary Figure S3). This suggests that compensation for the loss of *gamma*-syn function by other synuclein(s) exerts much-more-profound effects on EEG spectral profiles, i.e., elevation of *beta* and suppression of *delta 2*, *theta* and *alpha* bands, when DA signalling has been activated (Supplementary Figure S3). This effect was also obvious when APO-induced changes in EEG spectral profiles observed in synuclein KO mice were compared with changes observed in WT mice, i.e., elevation of *beta*, particularly *beta 2*, and the suppression of higher frequency bands, particularly *alpha* (Supplementary Figure S4). Taken together, these observations again point to an importance of a balance of synucleins for neuronal function.

## 5. Conclusions

We found that changes in the composition of synucleins significantly affect EEG oscillation profiles in all studied areas of the nervous system and that the activation of DA signalling by APO treatment causes further genotype- and brain area–specific alterations in these profiles. Further studies should unveil molecular and cellular mechanisms linking a disbalance of synucleins and changes in the electrical activity of the brain, as well as whether and how EEG spectral analyses can be applied for the early differential diagnostics of synucleinopathies.

## Data Availability

Data are contained in the current article and its supplementary material.

## Supplementary Materials

The following supporting information can be downloaded at https://www.mdpi.com/article/10.3390/biomedicines10123128/s1, Figure S1—verification of the position of the electrode tip (arrows) following electrocoagulation of surrounding tissues; Figure S2—baseline changes of EEG recordings from four brain areas of synuclein KO mice compared with WT mice.; Figure S3—APO-induced changes of EEG recordings from four brain areas of synuclein KO and WT mice; Figure S4—APO-induced changes in EEG recordings from four brain areas of synuclein KO compared with APO-induced changes in WT mice.
