# Supplementary material for: Loss of the Synuclein Family Members Differentially Affects Baseline- and Apomorphine-Associated EEG Determinants in Single-, Double- and Triple-Knockout Mice
Source: Biomedicines. 2022 Dec 4;10(12):3128. doi: 10.3390/biomedicines10123128 (PMC9775760; doi:10.3390/biomedicines10123128)
Supplement: Supplementary file 1 [file biomedicines-10-03128-s001.zip › Supplementary Figure S1.pdf]

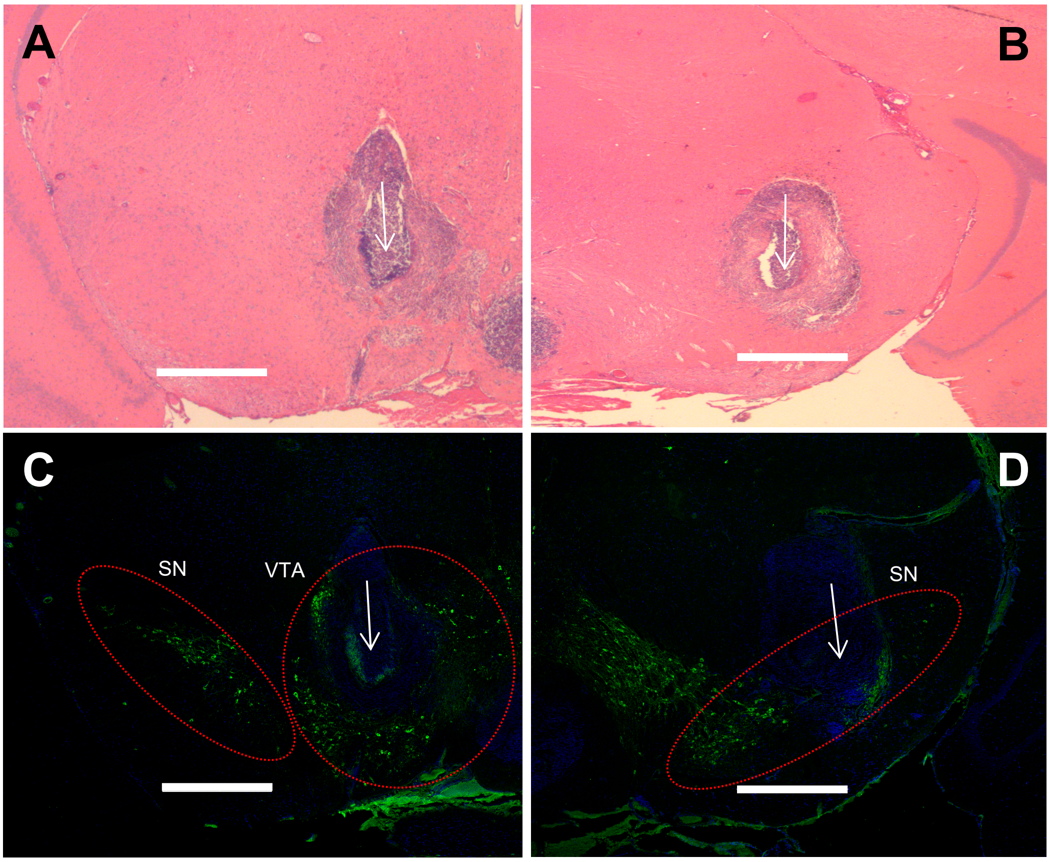

Verification of the position of the electrode tip (arrows) following electrocoagulation of surrounding tissues. Coronal sections of the mouse brain at the level of the ventral tegmental area (VTA) and substantia nigra (SN) stained with hematoxylin and eosin (**A**,**B**) or immunostained with antibody against tyrosine hydroxylase (TH) for detection of dopaminergic neurons (green signal in **C**,**D**). Scale bar, 500 μm.
