# Supplementary material for: Loss of the Synuclein Family Members Differentially Affects Baseline- and Apomorphine-Associated EEG Determinants in Single-, Double- and Triple-Knockout Mice
Source: Biomedicines. 2022 Dec 4;10(12):3128. doi: 10.3390/biomedicines10123128 (PMC9775760; doi:10.3390/biomedicines10123128)

Apomorphine-induced changes in average EEG amplitude comparing to changes in WT animals

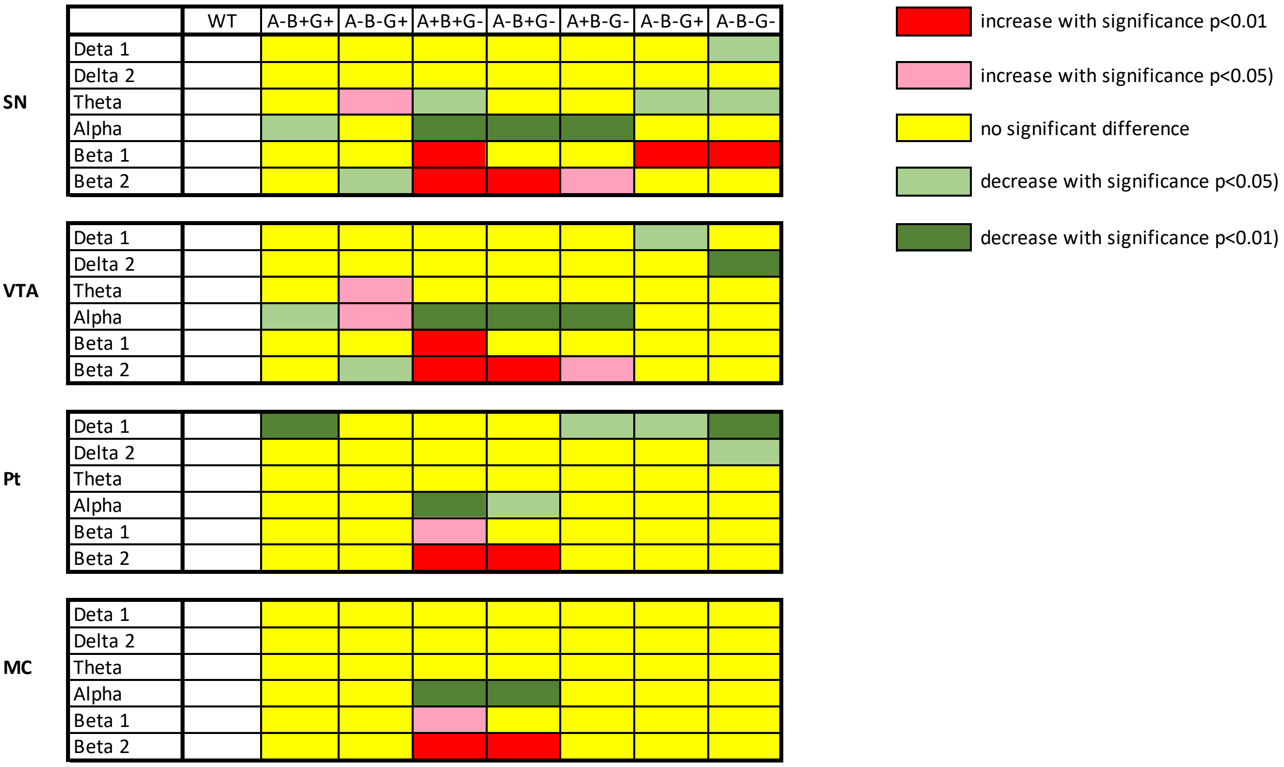

Supplement: Supplementary file 1 [file biomedicines-10-03128-s001.zip › Supplementary Figure S4.pdf]
